# Pathogenomic Inference of Virulence-Associated Genes in *Leptospira interrogans*

**DOI:** 10.1371/journal.pntd.0002468

**Published:** 2013-10-03

**Authors:** Jason S. Lehmann, Derrick E. Fouts, Daniel H. Haft, Anthony P. Cannella, Jessica N. Ricaldi, Lauren Brinkac, Derek Harkins, Scott Durkin, Ravi Sanka, Granger Sutton, Angelo Moreno, Joseph M. Vinetz, Michael A. Matthias

**Affiliations:** 1 Division of Infectious Diseases, Department of Medicine, University of California San Diego School of Medicine, La Jolla, California, United States of America; 2 J. Craig Venter Institute, Rockville, Maryland, United States of America; University of Tennessee, United States of America

## Abstract

Leptospirosis is a globally important, neglected zoonotic infection caused by spirochetes of the genus *Leptospira*. Since genetic transformation remains technically limited for pathogenic *Leptospira*, a systems biology pathogenomic approach was used to infer leptospiral virulence genes by whole genome comparison of culture-attenuated *Leptospira interrogans* serovar Lai with its virulent, isogenic parent. Among the 11 pathogen-specific protein-coding genes in which non-synonymous mutations were found, a putative soluble adenylate cyclase with host cell cAMP-elevating activity, and two members of a previously unstudied ∼15 member paralogous gene family of unknown function were identified. This gene family was also uniquely found in the alpha-proteobacteria *Bartonella bacilliformis* and *Bartonella australis* that are geographically restricted to the Andes and Australia, respectively. How the pathogenic *Leptospira* and these two *Bartonella* species came to share this expanded gene family remains an evolutionary mystery. *In vivo* expression analyses demonstrated up-regulation of 10/11 *Leptospira* genes identified in the attenuation screen, and profound *in vivo*, tissue-specific up-regulation by members of the paralogous gene family, suggesting a direct role in virulence and host-pathogen interactions. The pathogenomic experimental design here is generalizable as a functional systems biology approach to studying bacterial pathogenesis and virulence and should encourage similar experimental studies of other pathogens.

## Author Summary

Leptospirosis is one of the most common diseases transmitted by animals worldwide. It is important because it causes an often lethal febrile illnesses in tropical and subtropical areas associated with poor sanitation and agriculture. Leptospirosis may be epidemic, associated with natural disasters and flooding, or endemic in tropical regions. It is unknown how *Leptospira* cause disease and why different strains cause different severity of illness. In this study we attenuated (weakened) a highly virulent strain of *L. interrogans* by culturing it *in vitro* over several months. Comparison of the whole genome sequence before and after the attenuation process revealed a small set of genes that were mutated, and therefore associated with virulence. We discovered a putative soluble adenylate cyclase with host cell cAMP elevating activity, with implications for immune evasion and a new gene family that is upregulated *in vivo* during acute hamster infection. Interestingly, both *Bartonella bacilliformis* and *Bartonella australis* also have this unique gene family we describe in pathogenic *Leptospira*. This information aids in our understanding of *Leptospira* evolution and pathogenesis.

## Introduction

**Figure 1:**
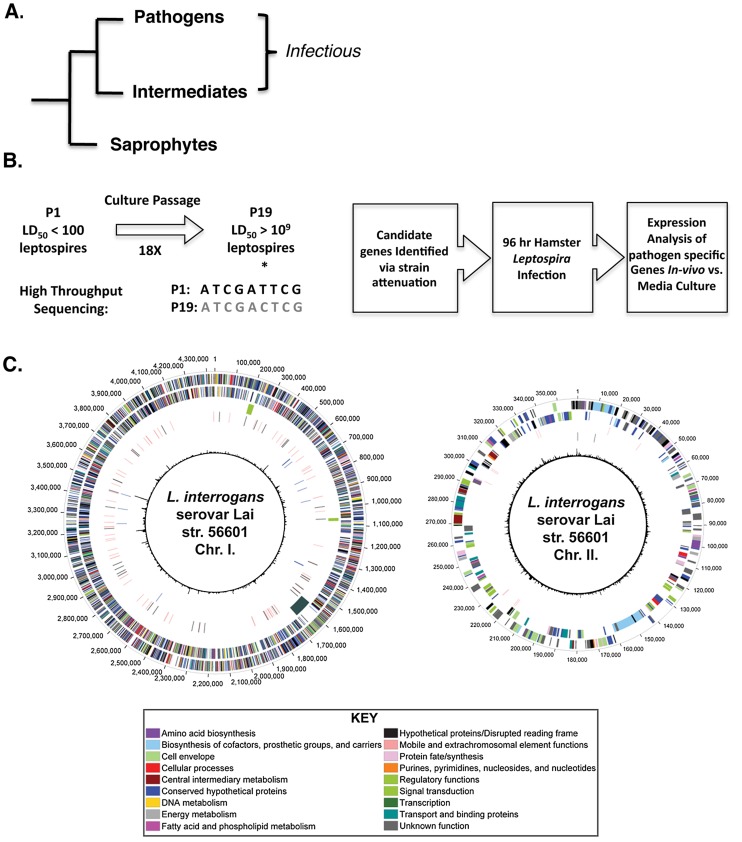
Pathogenomic analysis of *Leptospira interrogans* serovar Lai strain 556021 to identify virulence related genes. (**A**) Schematic of phylogenetic relatedness of “Pathogenic” (P), “Intermediate” (I) and “Saprophytic” (S) members of the genus *Leptospira*. (**B**) Workflow to identify putative virulence-associated genes. Asterisk denotes a hypothetical position in which a SNV has been identified (**C**) Genomic Locations of SNPs and PF07598 paralogs in the reference genome of *L. interrogans* serovar Lai strain 56601. Each concentric circle represents genomic data and is numbered from the outermost to the innermost circle. The outermost circles represent the predicted CDS on the + and − strands, respectively, colored by functional role categories (see key). The following circle descriptions apply to chromosome I. The third circle notes the location of predicted prophage regions (olive) and the LPS region (slate). The fourth circle indicates those CDS found to have non-synonymous amino acid substitutions (black) as well as the location of CDS annotated as “transposase” in Genbank (salmon). The fifth circle represents the location of the 12 PF07598 family members (blue). The innermost circle denotes atypical regions (χ^2^ value). For chromosome II, the outermost and innermost circles are the same as for chromosome I; however, the third circle notes the location of transposases (salmon), while the fourth circle indicates the location of the CDS found to have non-synonymous amino acid substitutions (black).

Leptospirosis, caused by spirochete bacteria of the genus *Leptospira*, is a zoonotic disease of high public health impact [1]. Globally, nearly 900,000 people are infected annually through contact with contaminated water, infected tissue or urine of mammalian reservoir hosts [2]. Phylogenetic analyses have resolved the genus into 3 distinct lineages, which are the focus of a pan-*Leptospira* genome project supported by the NIAID Genome Sequencing Center: nine pathogenic species; five intermediate species (eg. *L. fainei, L. licerasiae*); and six non-infectious saprophytic species (i.e. *L. biflexa*) (Fig. 1A) [3]–[6]. The greatest burden of disease is caused by the pathogenic species, mainly affecting people living in poverty and with poor sanitation [1], [2], [7]. Epidemics of leptospirosis associated with floods, monsoons, or hurricanes have a high morbidity and mortality with case fatality rates ranging as high as 20–25% in hospitalized patients leading to refractory shock, jaundice, renal failure, and pulmonary hemorrhage [1].

Despite its severity and global importance, the molecular pathogenesis of leptospirosis remains poorly understood [8]. *Leptospira* penetrate mucosal epithelium and damaged integument then hematogenously disseminate to localize within multiple organs, including the liver and kidney, within 72 hours. Leptospiremia may continue for up to two weeks after onset of symptoms with blood bacterial concentrations reaching as high as 10^6^–10^7^ organisms/mL in infected patients [9], [10]. The only virulence factor genetically defined to date is the surface lipoprotein Loa22 [11], but mechanisms by which it contributes to disease pathogenesis remain unknown. Other virulence-associated genes include heme oxygenase [12], LPS [13], clpB [14], and flagellar components [15], [16]. Although random transposon mutagenesis has been used to identify a few putative leptospiral virulence-related genes [17] [18], further progress has been hindered by the lack of efficient gene-targeted mutagenesis techniques in pathogenic *Leptospira* [8].

We used a functional systems biology (pathogenomic) approach to identify candidate virulence genes, by genomic comparison of a culture-attenuated *Leptospira interrogans* serovar Lai strain 56601 (LD_50_>10^9^)(Fig. 1B) with its virulent, isogenic parent (LD_50_<100) [19]. *In vivo* relevance of identified candidate genes was determined by quantification of expression of candidate genes on day 4 after hamster infection in blood, liver, and kidney compared to *in vitro* culture.

## Materials and Methods

### Ethics statement

This study was carried out in accordance with the recommendations in the Guide for the Care and Use of Laboratory Animals of the National Institutes of Health in AAALAC-approved facilities. The experimental animal work was approved by the Institutional Animal Care and Use Committee of the University of California San Diego under protocol S03128H.

### Bacterial strain maintenance and attenuation of *L. interrogans* serovar Lai strain 56601

All strains were maintained *in vitro* in Ellinghausen-McCullough-Johnson -Harris (EMJH) media using standard protocols and are available from BEI Resources. *L. interrogans* serovar Lai strain 56601 was obtained from Dr. David Haake (UCLA). Virulence was selected for by serial passage through hamsters so that the P1 strain used in the present study had an LD_50_ of ∼10 organisms. *L. interrogans* serovar Lai strain 56601_P1 was attenuated by 18 bi-weekly subcultures *in vitro*. Virulence was assessed every five to ten subcultures using three-week-old male Golden Syrian Hamsters. Following a final subculture, genomic DNA was prepared from this attenuated strain (designated P19) on which next generation sequencing was carried out.

### Genome assembly of virulent P1 and attenuated P19 *L. interrogans* serovar Lai strain 56601 and non-synonymous SNV (nsSNV) detection

We generated 4,379,515 and 5,340,095 unpaired shotgun reads from *L. interrogans* serovar Lai 56601_P1 and 56601_P19, respectively using next generation sequencing technology. All reads were 36 bases long. Both genomes were assembled using the comparative assembler AMOScmp. The AMOSCmp-shortReads-alignmentTrimmed pipeline that runs within AMOScmp, was used to look for exact matches of each read to the published *L. interrogans* serovar Lai 56601 genome of at least 20 bp, permitting a maximum consensus error rate of 0.06% (i.e. at most two mismatches in any read). This script runs a reference-based trimming of the 3′-end of the reads prior to assembly. We found that trimming of at most 4 bases from the 3′-end of the reads based on their matches to the reference produced better assemblies than un-trimmed reads. The P1 assembly used 3,919,609 reads, leaving 459,906 unassembled singletons, while the P19 assembly used 4,915,295 leaving 424,800 singleton reads. The 56601_P1 genome was assembled into 167 contigs with an average length of 28,124 kb and an N50 length of 105,604 kb and the P19 genome into 97 contigs, average length 48,417 and N50 of 190,406. We checked the quality of both assemblies using the amosvalidate pipeline, which runs within AMOScmp. This pipeline identifies misassembly features such as increased read depth and correlated SNVs (i.e. one or more reads with the same SNV, which is unlikely to be due to sequencing error), both indicative of collapsed repeats. We found that both assemblies were high quality with at most 5 potential misassembly features in longer contigs. These potential misassemblies were inspected manually using the Hawkeye viewer and reassembled if necessary using minimus, which employs a stricter assembly algorithm. The unfinished 56601_P1a and 56601_P19 genomes were aligned and SNVs identified using the MUMmer v3.22 software package.

### RT-qPCR in vivo gene expression analysis

Three wk old Golden Syrian Hamsters were infected via intraperitoneal injection with 10^8^ low passage *L. interrogans* serovar Lai strain 56601. 96 hours post infection total RNA was collected using TRIzol (Invitrogen) from blood, liver, and kidney tissue, as well as from a 96-hour EMJH culture of *L. interrogans* grown at 30°C. Total RNA was reverse transcribed using a QuantiTect reverse transcription kit (Qiagen). cDNA was amplified using a CFX96 thermal cycler (Bio-Rad) using PerfeCta SYBR Green FastMix (Quanta Biosciences). PCR was carried out at 95°C for 3 min, a touchdown gradient of 14 cycles of (94°C 10 s, 80°C 45 s) decreasing 1°C/cycle, followed by 40 cycles of (94°C 30 s, 65°C 45 s). Ct values were normalized to the leptospiral 16S rRNA gene and expression fold change calculated using the Pfaffl method [20]. Primer sequences are listed in Table S3 in Text S1.

### Domain architecture analysis of LA_4008 and other related AGC proteins

Domain architecture comparison of LA_4008 with orthologs of *Myxococcus xanthus*, *Corallococcus coralloides*, *Stigmatella aurantiaca*, and *Mycobacterium tuberculosis* using NCBI CD Search, SMART, and TPRPred. Protein homology analysis was carried out using BLAST using the following reference sequences: LA_4008 (NP_714188.1), MXAN_4545 (YP_632713.1), COCOR_04748 (YP_005370712.1), STAUR_4866 (YP_003954471.1), Rv0386 (CCP43116). The coverage for the query sequence, statistical significance (E-value), and maximum amino acid identify (“Max Ident”) are indicated at right for each predicted primary sequence. Identified domains were then graphically represented using the DOG 1.0 program (http://dog.biocuckoo.org)

### *Leptospira* Concentrated Culture Supernatant (CCS)

*L. interrogans* Lai 56601 or *L. licerasiae* Varillal were grown in EMJH media +10% heat inactivated rabbit serum at 37°C on a rotating shaker for 96 hr. Culture was centrifuged for 30 min at 10,000× g. Supernatant was decanted, filtered through a .22 µm syringe filter unit (Millipore), and concentrated 10× in an Amicon Ultra 10K MWCO centrifugal filter unit (Millipore). No leptospires were observed in the CCS after concentration using darkfield microscopy.

### CCS cAMP elevating activity

CCS was incubated with monolayers of THP-1,a human monocyte/macrophage cell line. At 4, 6, and 20 hours cells were rinsed 3× in PBS and analyzed for cAMP (Direct cAMP EIA kit, Enzo Life Sciences). Secondly, CCS from *L. interrogans* Lai and *L. licerasiae* were incubated with THP-1 monolayers for 4 hours, and assayed for cAMP.

### CCS immunodepletion studies

Rabbits were used to generate anti-peptide antisera against LA_4008 using a protein specific, sixteen amino acid fragment (SVEEDPLTREIDRKQK) conjugated to keyhole limpet hemocyanin as a carrier protein (Pacific Immunology, Ramona, CA). The IgG fractions from pre-immunization and production bleeds were purified using a Melon Gel IgG Purification kit (Thermo Scientific) and covalently linked to magnetic beads using a NanoLink BeadLink Kit (Solulink). Antibody linked beads were incubated with CCS overnight at 4°C on a rotating shaker. Beads were separated on a QuadroMACS separation unit (Miltenyi Biotec). Depleted CCS was applied to THP-1 monolayers and incubated for 4 hours. Cells were rinsed 3× in PBS and analyzed for total cAMP using the Direct cAMP EIA kit.

### Phylogenetic analysis of PF07598 paralogous protein family

*L. interrogans*, *L. borgpetersenii* and *B. bacilliformis* full-length sequences were downloaded from the Uniprot databa se (http://www.uniprot.com) and aligned using MAFFT v7 (http://mafft.cbrc.jp/alignment/software) with default parameters. The evolutionary history was inferred by using the Maximum Likelihood method based on the Whelan and Goldman frequency model [21]. Statistical support of the tree topology was obtained from 500 bootstrap replicates. A discrete Gamma distribution was used to model evolutionary rate differences among sites. The rate variation model allowed for some sites to be evolutionarily invariable. The tree is drawn to scale, with branch lengths measured in the number of substitutions per site. All positions containing gaps and missing data were eliminated. There were a total of 271 positions in the final dataset. Evolutionary analyses were conducted in MEGA5 [22].

### Pan-genomic analysis of attenuated genes and PF07598 orthologs

The genomic data analyzed here are publically available and are from newly generated, unpublished *Leptospira* whole genome sequence data produced by the JCVI as part of the white paper “*Leptospira* Genomics and Human Health,” sponsored by the NIAID-funded Genome Sequencing Centers. PanOCT [23] was run using default settings with the exception that a dynamically determined pairwise cutoff was implemented, not available in the current release, but available upon request. The following genomes, representing all 20 *Leptospira* spp. were used: *L. alexanderi* sv. Manha 3 str. L 60^T^ (Genbank:AHMT00000000), *L. alstoni* sv. Pingchang str. 80-412 (Genbank:AOHD00000000), *L. biflexa* sv. Patoc str. Patoc I Paris (Genbank:CP000786), *L. borgpetersenii* sv. Javanica str. UI 09931 (Genbank:AHNP00000000), *L. broomii* sv. undetermined str. 5399^T^ (Genbank:AHMO00000000), *L. fainei* sv. Hurstbridge str. BUT 6^T^ (Genbank:AKWZ00000000), *L. inadai* sv. Lyme str. 10^T^ (Genbank:AHMM00000000), *L. interrogans* sv. Copenhageni str. Fiocruz L1-130 (Genbank:AE016823), *L. interrogans* sv. Copenhageni str. M20 (Genbank:AOGV00000000), *L. interrogans* sv. Lai str. 56601 (Genbank:AE010300), *L. kirschneri* sv. Cynopteri str. 3522 C^T^ (Genbank:AHMN00000000), *L. kmetyi* sv. undetermined str. Bejo-Iso9^T^ (Genbank:AHMP00000000), *L. licerasiae* sv. Varillal str. VAR 010^T^ (Genbank:AHOO00000000), *L. meyeri* sv. Hardjo str. Went 5 (Genbank:AKXE00000000), *L. noguchii* sv. Panama str. CZ 214^T^ (Genbank: AKWY00000000), *L. santarosai* sv. Shermani str. 1342K^T^ (AOHB00000000), *L. terpstrae* sv. Hualin str. LT 11-33^T^ (Genbank: AOGW00000000), *L. vanthielii* sv. Holland str. WaZ Holland (Genbank:AOGY00000000), *L. weilii* sv. Ranarum str. ICF^T^ (Genbank:AOHC00000000), *L. wolbachii* sv. Codice str. CDC (Genbank:AOGZ00000000), *L. wolffii* sv. undetermined str. Khorat-H2^T^ (Genbank:AKWX00000000), *L. yanagawae* sv. Saopaulo str. Sao Paulo^T^ (Genbank:AOGX00000000).

### Statistics

Data were analyzed using GraphPad Prism 5.0. Significance was assessed using one-way ANOVA followed by Tukey's HSD post hoc testing. P-values are reported as *** = p<0.001, ** = p<0.01, * = p<0.05.

## Results

### Pathogenomic identification of protein coding genes in *Leptospira interrogans* serovar Lai and patterns of tissue-specific up-regulation in vivo

Comparison of the wild type and attenuated *L. interrogans* Lai 55601 genomes identified 41 non-synonymous single nucleotide variants (nsSNVs) in a total of 35 protein-coding genes (CDS; Table S1 in in Text S1). P19 sequence analysis revealed that all SNVs were homogeneous within the culture population; minority populations were not detected at the limit of detection of the Illumina sequencing platform (<4%). For the purposes of this study, therefore, the bacterial populations were considered clonal.

**Figure 2:**
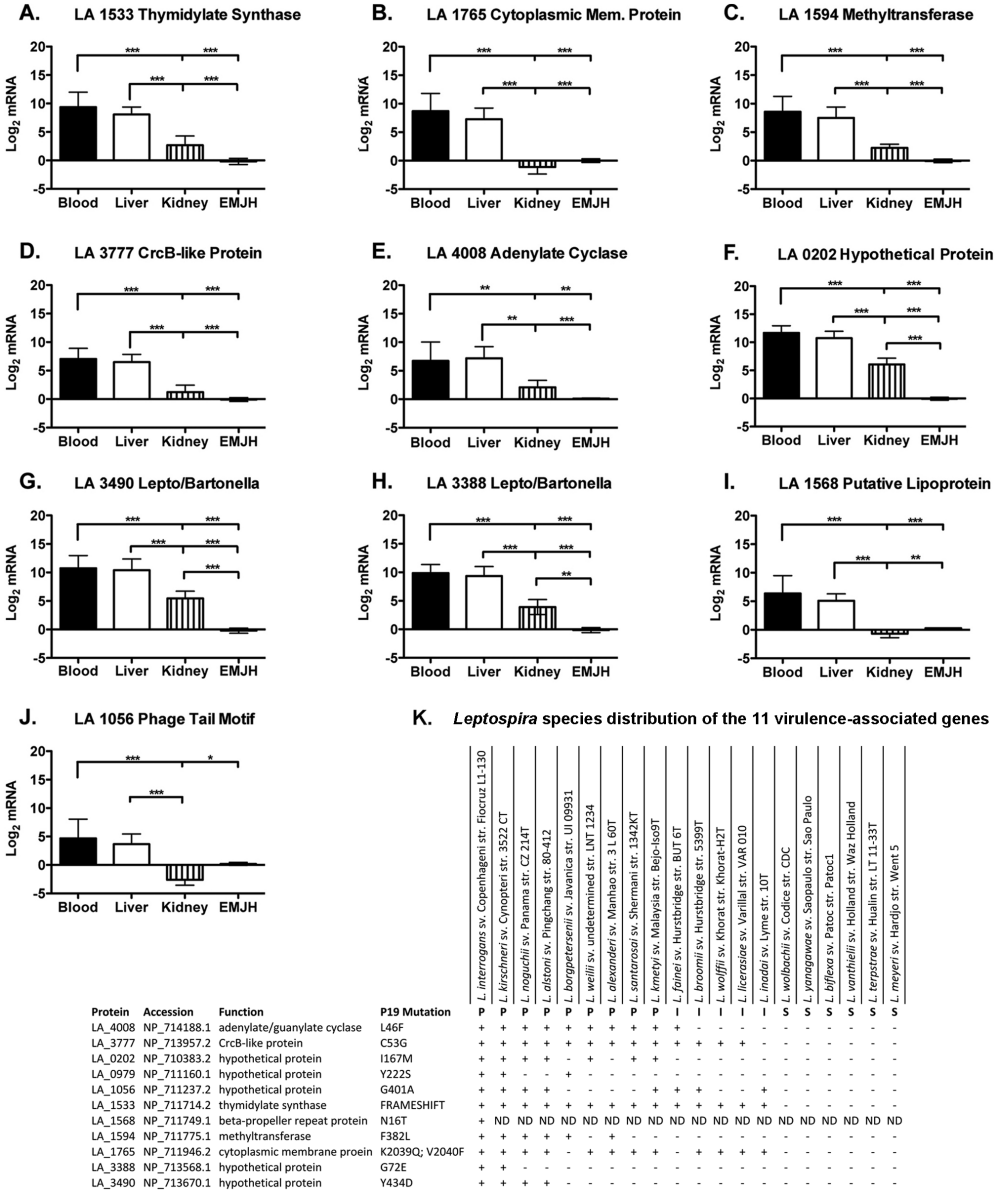
*In vivo* transcriptional analysis of putative virulence-associated genes. *In vivo* relevance of the identified virulence-related genes, mRNA transcript levels of the genes identified by the pathogenomics approach was assessed by real time, reverse transcriptase quantitative PCR of blood, liver and kidney 4 d after hamster infection, compared to log phase *in vitro* cultured *Leptospira*. Leptospiral gene expression levels in infected tissue vs. EMJH were expressed logarithmically as the log_2_ of the fold change between the two conditions (**A–J**). 16S rRNA transcript levels (previously validated [61]) were used to normalize gene expression in tissues and under the different conditions (Fig. S1 in Text S1). Expression of 10/11 identified genes was detectable *in vivo* in all three tissues assayed; the exception was the hypothetical protein LA_0979. The remaining 10 genes were detected in all three tissues assayed. Expression varied between groups of animals, and interestingly, the highest levels of up-regulation were found in leptospires isolated from the blood of infected animals, with transcript levels also being up in bacteria from the liver. Virulence-associated genes were variably up-regulated in kidney. The data represented are the mean ± SEM of 3 independent experiments (n = 7 animals). (**K**) *Leptospira* species distribution of the 11 virulence-associated genes identified and associated single nucleotide variants found in coding sequences of the avirulent passage (P19) strain. Protein code is according to the annotated protein database; Accession is the GenBank code for the protein.

Filtering to include CDS restricted to pathogenic *Leptospira* species identified 11 genes (Fig. 2K). These CDS are highly conserved among pathogenic *Leptospira* species (Fig. 2K). *In vivo* transcriptional analysis identified that of these 11 pathogen-specific genes, 10 were up-regulated *in vivo* during acute hamster infection (Fig. 2, normalized to the 16S rDNA gene, Fig. S1 in Text S1). Transcriptional up-regulation of CDS was as high as several thousand-fold, with a much higher dynamic range than found with *in vitro* conditions used in previously reported systems biology analyses (summarized in Table S2 in Text S1).

### Identification of a putative leptospiral protein with host cAMP elevating activity

**Figure 3:**
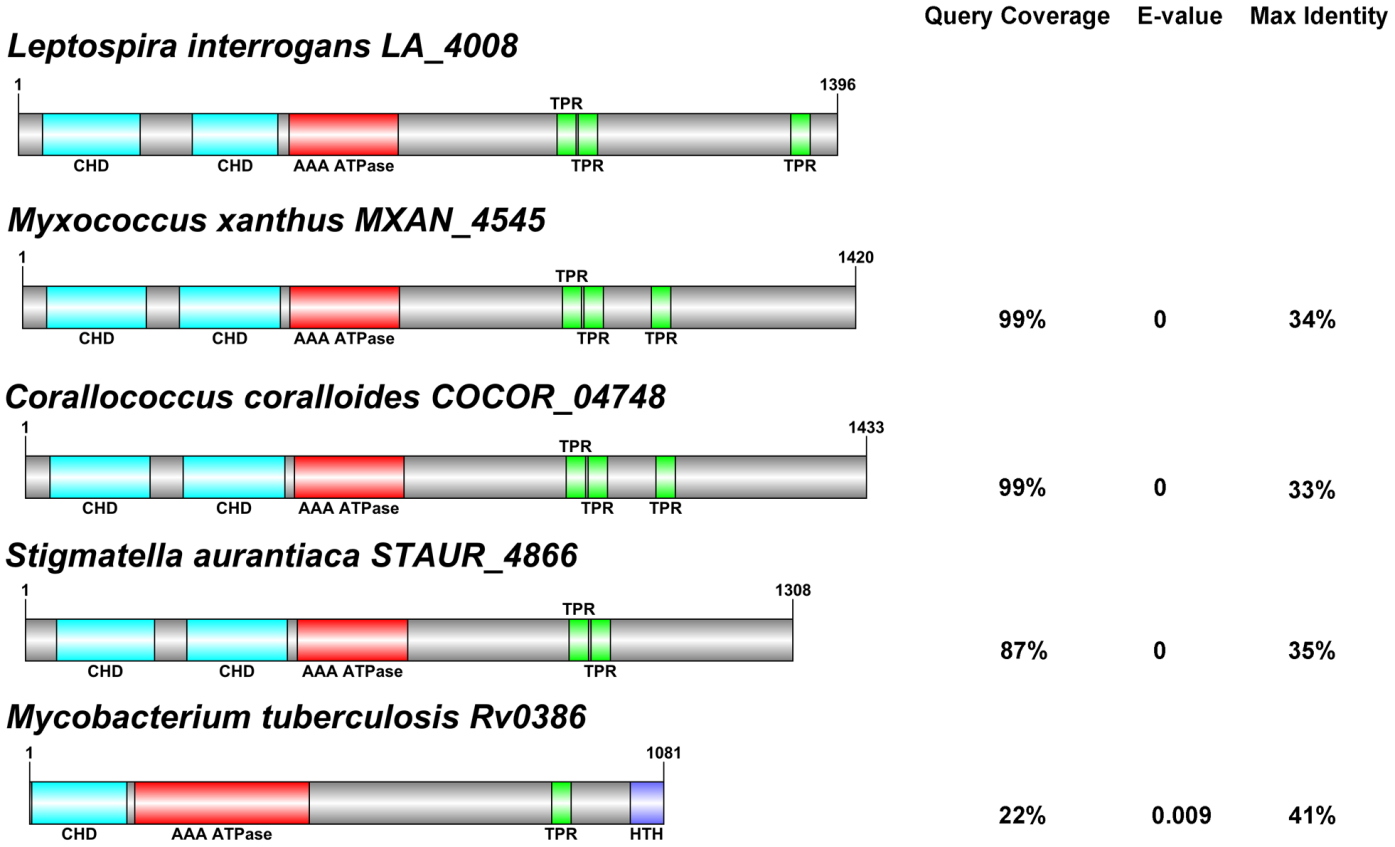
Ortholog sequence analysis of pathogenic *Leptospira* adenylate/guanylate cyclase compared to predatory environmental bacteria and the pathogen, *Mycobacterium tuberculosis*. Domain architecture comparison of LA_4008 with orthologs of *Myxococcus xanthus*, *Corallococcus coralloides*, *Stigmatella aurantiaca*, and *Mycobacterium tuberculosis* using NCBI CD Search, SMART, and TPRPred. Protein homology analysis was carried out using BLAST using the following reference sequences: LA_4008 (NP_714188.1), MXAN_4545 (YP_632713.1), COCOR_04748 (YP_005370712.1), STAUR_4866 (YP_003954471.1), Rv0386 (CCP43116). The coverage for the query sequence, statistical significance (E-value), and maximum amino acid identify (“Max Ident”) are indicated at right for each predicted primary sequence.

**Figure 4:**
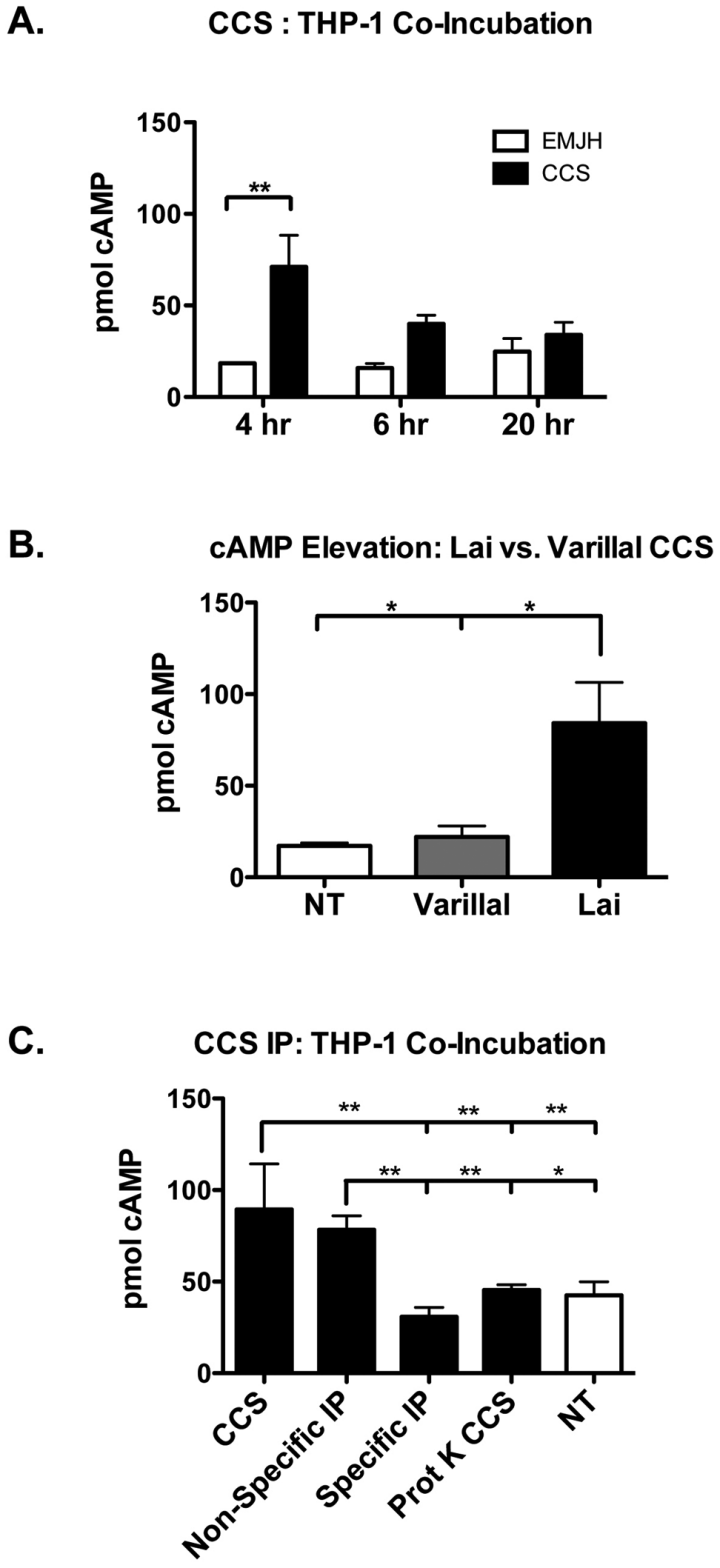
Confirmation of cAMP induction in target mammalian cells by LA_4008 activity in leptospiral culture supernatant. (**A**) THP-1 cell monolayers were treated with leptospire-free concentrated culture supernatant (CCS) from *L. interrogans* Lai or EMJH negative control. (**B**) THP-1 monolayers were treated with CCS from *L. interrogans* Lai or *L. licerasiae* Varillal, NT = not treated. (**C**) THP-1 cell monolayers were treated with CCS, CCS that was immunoprecipitated (IP) with specific anti-peptide antibody raised in rabbits and non-specific anti-LA 4008 antibody, and CCS that was digested with proteinase K. Values in all experiments are represented as the mean (n = 3) ± SD.

Of particular interest is LA_4008, a putative adenylate/guanylate cyclase (AGC) that lacks transmembrane helices typical of integral membrane cyclases involved in signal transduction, suggesting that this protein may be soluble. While another adenylate/guanylate cyclase was found in our screen in pathogens and intermediates (Table S1 in Text S1; LA_0027), this protein is predicted to be a housekeeping gene, a membrane- bound and intracellular, and not likely to be found in the extracellular milieu. Orthologs of LA_4008 are found only in pathogenic *Leptospira* and the intermediately pathogenic strain *L. fainei*, Fig. 2K. Other bacterial adenylate cyclases lacking transmembrane domains include the soluble cyclase class of toxins of the pathogens *Mycobacterium tuberculosis, Bordetella pertussis, Bacillus anthracis, Yersinia pestis, and Pseudomonas aeruginosa* which modulate host cellular responses to infection [24]. Sequence analysis by SMART, TPRPred, and NCBI conserved domain (CD) search revealed a unique domain architecture for LA_4008 consisting of two tandem N-terminal class III cyclase homology domains followed immediately by an AAA-ATPase domain, and finally a series of C-terminal tetratricopeptide (TPR) domains (Fig. 3), that are known to mediate protein-protein interactions and have recently been recognized as components of bacterial virulence mechanisms [25], [26]. LA_4008 also shares striking homology to a toxin (NCBI protein cluster PCLA_814229) shared by predatory species of the δ-proteobacterial order Myxococcales (Fig. 3). The domain structure shared by this protein cluster is reminiscent of another pathogenesis-related adenylate cyclase, Rv0386 of *Mycobacterium tuberculosis*. This domain structure, with precedent in both pathogenic and environmental bacteria, has been experimentally shown to increase cyclic AMP levels in host macrophages and impair the innate immune response to infection (Fig. 3), [27], [28]. To test whether LA_4008 has the potential to elevate cyclic AMP in host cells, concentrated *L. interrogans* serovar Lai strain 56601 EMJH culture supernatant (CCS) was added to *in vitro* monolayer cultures of macrophage-like THP-1 cells and the cells were washed and lysed at various times over 20 hr, and intracellular cyclic AMP levels were assayed. All CCS preparations were microscopically confirmed to be absent of visible leptospires prior to all experiments. We observed that a *L. interrogans*-derived soluble factor from culture supernatant stimulated a transient rise in intracellular macrophage cAMP levels, peaking at four hours (Fig. 4A). Next, the cAMP elevating activity of CCS was compared between *L. interrogans* serovar Lai (which has LA_4008), and the intermediate *L. licerasiae* serovar Varillal (Fig. 4B) (which does not have an ortholog of LA_4008). The results of this experiment was consistent with the hypothesis that cAMP elevating activity may be related to pathogenic *Leptospira* species containing the LA_4008 AGC but not by intermediate *Leptospira* (Fig. 1A), and therefore is not a general feature of all infectious leptospires. To further test if LA_4008 is responsible for the elevated target cell cAMP, CCS was digested with proteinase K prior to addition to THP-1 cells (important because some bacterial LPS can also elevate cAMP levels in host cells [29]) and, more critically, immune-depleted with a specific anti-LA_4008 antibody before adding the CCS to THP-1 cells. As a control, CCS was also immune-depleted with pre-immune serum of the host animal in which the anti-LA_4008 antiserum was generated. Both protease treatment and specific immunodepletion, but not non-specific depletion blocked CCS-mediated increases in intracellular cAMP levels in THP-1 cells (Fig. 4C), consistent with the hypothesis that LA_4008 from *L. interrogans* Lai is a cAMP-elevating factor in host cells.

### Identification of a paralogous gene family shared by pathogenic *Leptospira*, *Bartonella bacilliformis*, and *Bartonella australis* with profound, tissue-specific up-regulation in vivo in an acute leptospirosis infection model in hamsters

**Figure 5:**
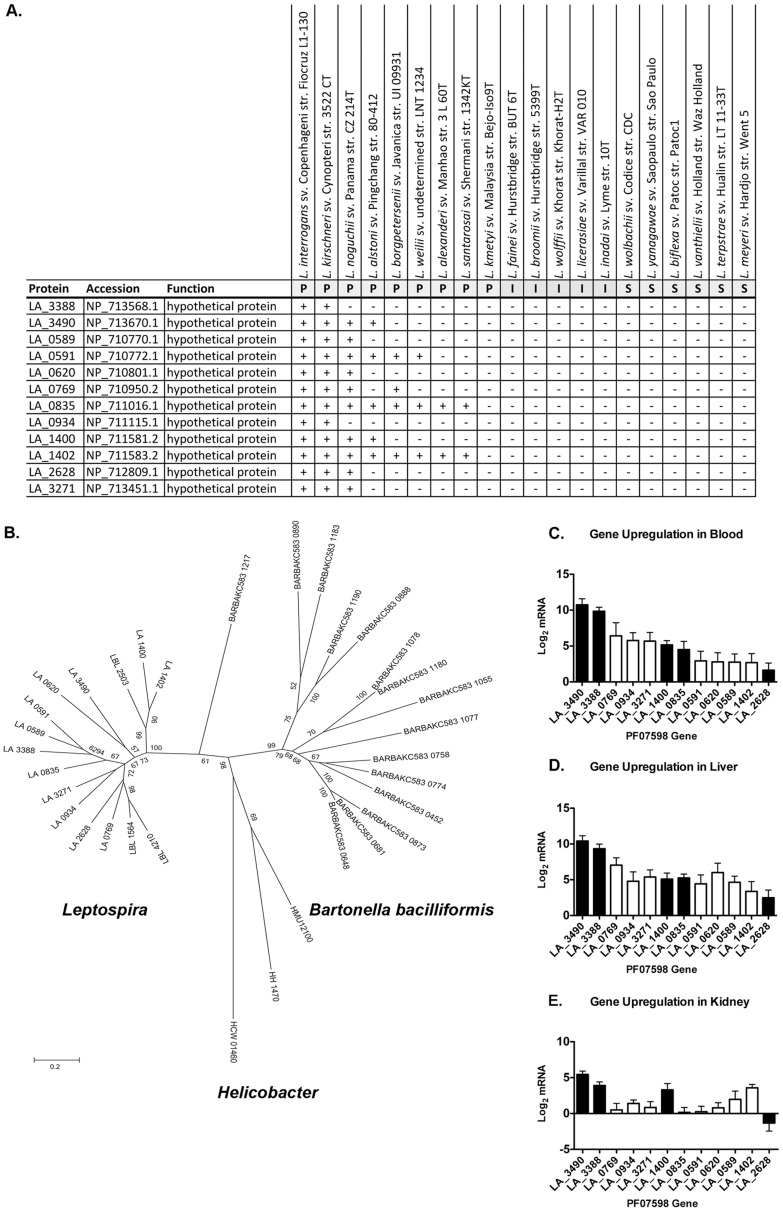
Phylogenetic and in vivo gene expression analysis of the PF07598 paralogous gene family shared by pathogenic *Leptospira* and *Bartonella bacilliformis*. (**A**) Distribution of the paralogous gene family shared by *Leptospira* and *Bartonella bacilliformis* in the genus *Leptospira*. P, pathogen; I, intermediate; S, saprophyte. (**B**) An unrooted phylogenetic tree was constructed of protein sequences from all identifiable homologs of the DUF1561 protein family found in GenBank and the PATRIC databases, which included predicted sequences from the following bacteria (*Helicobacter* spp. and *B. bacilliformis* genome locus tags and protein sequences used for constructing the tree are listed in Table S4 in Text S1): *L. interrogans* Lai, *L. borgpeterseni* Hardjo; *Helicobacter cetorum*, *H. hepaticus* and *H. mustelae*; and *B. bacilliformis* full-length sequences were aligned using MAFFT. Node labels represent support from 500 bootstrap replicates. Tree drawn to scale, with branch lengths measured in the number of substitutions per site. All positions containing gaps and missing data were eliminated. Analyses were conducted in MEGA5. (**C–E**) *In vivo* relevance of the leptospiral paralogous gene family was assessed in the acute hamster infection model as described in Fig. 1. Transcript levels of the genes were assessed by real time, reverse transcriptase quantitative PCR of blood, liver and kidney 4 days after hamster infection and compared to log phase *in vitro* cultured *Leptospira*. Leptospiral gene expression levels in infected tissue vs. EMJH medium alone were expressed logarithmically as the log_2_ of the fold change between the two conditions. Solid bars indicate proteins containing predicted signal peptides that suggest extracellular presence, i.e. secretion or cell-surface, of the protein, consistent with bacterial interaction with the host. Data represented are the mean ± SEM of 3 independent experiments (n = 7 animals).

During our analysis of attenuation mutations we identified two members (LA_3490, LA_3388) of a newly discovered paralogous gene family that is shared between pathogenic *Leptospira* but conspicuously absent in the intermediate and saprophytic species. All full-length members of this family (PF07598/DUF1561) are predicted to have secretory signal peptides, although degenerate forms do occur. Past the signal peptide, Cys residues are invariant at twelve positions, and occur nowhere else, suggesting a conserved pattern of disulfide bond formation and implying extracellular function (Fig. S2 in Text S1). In a given genome, the most closely related paralogs are often tandem. Otherwise, gene neighborhood analysis provided no clue to protein function. Paralog counts in pathogenic *Leptospira* range from two in the leptospire *L. santarosai* to 12 in *L. kirschneri* serovar Cynopteri and *L. interrogans* (Fig. 5A). Interestingly the PF07598 gene family has also been recently described in the unrelated α-proteobacteria species *Bartonella bacilliformis* and *Bartonella australis*. *B. bacilliformis* has 15 paralogs in its genome with *B. australis* having nearly the same (Fig. 5B) [30]. In addition single gene copies were found in three animal-infecting **ε**-proteobacteria, *Helicobacter hepaticus*, *H. mustelae*, and *H. cetorum*.

There are great phylogenetic distances separating the genera that contain this gene family, but paralogs are restricted to select animal-infecting species within each lineage; suggesting that these proteins may be uniquely related to host adaptation. All 12 members of the leptospiral PF07598 gene family were analyzed for *in vivo* expression in hamsters acutely infected with virulent, wild type *L. interrogans* Lai 55601. All members of this gene family were up-regulated in blood and liver to varying degrees, with LA_3490 and LA_3388, both containing secretory signal peptide sequences, being most highly up-regulated (more than ∼1000-fold); all members of this gene family were up-regulated in the circulation and liver to varying degrees. In contrast, up-regulation of other members this gene family significantly varied among experimental animals in kidney (Fig. 5C–E).

### Other pathogenomically-identified putative virulence genes in *Leptospira* spp

Other pathogenomically-associated virulence genes include the following:

**LA_1056**: This gene has two predicted transmembrane helices and shares a conserved PHA00965 domain with tail proteins found in Gram-positive bacteriophages. This protein shows similarity to phage tape measure proteins after repeated rounds PSI-BLAST. Recent studies involving the phage-encoded pblA in *Streptococcus mitis* have identified a sequence weakly reminiscent of a tape measure motif protein by PSI-BLAST as an adhesin-type molecule used for bacterial attachment to platelets [31]–[34].

**LA_1765**: This protein has similarity to spvB, a protein from a group of plasmid-encoded virulence genes that mediate lethal infection in nontyphoid *Salmonella* strains [35].

**LA_1533**: a flavin-dependent thymidylate synthase. This unusual and newly described class of enzyme is expressed by many clinically relevant pathogens, including *Bacillus anthracis*, *Borellia burgdorferi*, *Campylobacter jejuni*, *Clostridium difficile*, *Helicobacter pylori*, *Mycobacterium tuberculosis*, and *Treponema pallidum* during infection as part of an alternative thymidine synthesis pathway [36]–[38].

**LA_0202**: a gene of unknown function previously reported to be transcriptionally up-regulated in virulent *L. interrogans* Lai 55601 when compared to another avirulent strain [39].

**LA_1568**: a putative lipoprotein with β-propeller repeats that has not been previously studied. Lipoproteins are important mediators of spirochete virulence, with the *L. interrogans* genome encoding over one hundred lipoproteins [40], the function and localization of many remain unclear.

## Discussion

Here we describe the use of pathogenomics to identify novel potential virulence genes in the pathogenic spirochete *Leptospira interrogans*. Previous work to identify mechanisms of pathogenesis by gene knockouts and transposon mutagenesis has not yet yielded detailed mechanistic insights into the role of individual genes play in the pathogenesis of leptospirosis. It has long been known in the leptospirosis field that serial *in vitro* passage of pathogenic *Leptospira* yielded attenuated organisms; the converse, serial passage of liver homogenates of infected animals selects for virulence. A previous study explored the genomic and proteomic differences between a pathogenic *L. interrogans* serovar Lai strain 56601, and an avirulent strain IPAV [41]. These data must be carefully considered because the analyzed strains are not isogenic (the IPAV strain is of unknown provenance since details of its original isolation are unavailable) nor do they provide any *in vivo* relevance for identified genes, focusing instead on proteomic differences between strains during *in vitro* EMJH culture. Our current study, which employed whole genome sequence comparison of an attenuated strain with its isogenic pathogenic parent, yielded a small set of protein coding genes (CDS) with point mutations. While most of the 11 specific mutations found here cannot be quantitatively attributed to specific aspects of virulence or pathogenicity, our pathogenomic approach yielded the identification of a novel leptospiral AGC with cAMP elevating activity in host cells and a hitherto unstudied large gene family that is broadly up-regulated, in a tissue-specific manner, *in vivo* during an animal model of acute leptospirosis.

The identification of a non-transmembrane bound AGC in pathogenic *Leptospira* is particularly important for two reasons. First, the primary structure implies a non-housekeeping function since the protein is not predicted to be membrane-associated, unlike the housekeeping AGC. Second, the host cell cAMP elevating activity of LA_4008 reported in this study is the first demonstrated evidence of a possible biological mechanism that could contribute to virulence for *Leptospira*. Although long established and accepted as a virulence mechanism in other pathogens, the evidence of elevation of host cAMP levels by *L. interrogans* suggests a previously unknown mechanism of pathogenesis and immune evasion for this bacterium, especially given recent evidence that pathogenic *Leptospira* may reside within macrophages *in vivo* [42], [43]. Manipulation of intracellular cAMP levels in immune cells may be an important means of attenuating host responses to infection [44], an enticing hypothesis given the up-regulation of this gene upon leptospiral entry into the bloodstream observed in this study. Many human pathogens exploit host cell cAMP signaling during infection, for example, the pore-forming toxin CyaA of the respiratory pathogen *Bordetella pertussis* penetrates host cells where it catalyzes the unregulated conversion of cellular ATP to cAMP, thereby impairing superoxide production, chemotaxis, cytokine production, and phagocytosis [45]–[47]. Similar effects are caused by the edema factor (EF) of *Bacillus anthracis*, the ExoY toxin of *Pseudomonas aeruginosa*, and the AGC toxin of *Yersinia pestis* [24], [48]–[50]. Due to an unexpected loss of the cryogenically preserved stock cultures, we were unable to assess the cAMP elevating activity of the attenuated P19 strain. However, we would hypothesize that the attenuated SNV-containing variant could have either absolute elimination or quantitative reduction in cAMP elevating activity; this possibility will be addressed directly in ongoing experiments by quantifying the effect of recombinantly producing wild type and mutant LA_4008 on THP-1 and other target cells. We also believe that any observed reduction in activity would have been a quantitative not qualitative difference. Regardless, our findings demonstrate that LA_4008 contributes to a transitory increase in cAMP levels in host cells, and that further experiments are certainly needed to assess the functional consequences of cAMP intoxication in host immune cells during leptospirosis. To formally determine the role of LA_4008 in *Leptopira* pathogenesis is the subject of ongoing experiments, including determining whether this protein modulates mechanisms of evading host defenses.

The identification of a paralogous protein family shared by pathogenic *Leptospira* spp., and two α-proteobacteria *B. bacilliformis*, and *B. australis* was particularly intriguing. The observation that this gene family expanded in pathogenic *Leptospira* and the two *Bartonella* spp. suggests that ancestors of these pathogens must have co-existed at some time and place in the past. Phylogenetic analysis suggests a common origin of this gene family, and revealed a greater divergence in the *Bartonella* members, indicated by greater branch length differences (Fig. 5B). Regardless of the source of the primary ortholog, the founding gene was presumably transferred after the branching of pathogenic *Leptospira* from the other clades of *Leptospira*, although it is also possible that gene loss occurred in intermediates or saprophytes evolved from pathogens. Although we cannot speculate on the molecular mechanism of gene transfer, it is interesting to consider the conditions that would have been conducive to such an event. *L. interrogans* is a globally distributed bacterium that can infect many vertebrate hosts as well as live in the environment; it is considered an extracellular parasite, although evidence is mounting that *Leptospira* [42], [43], [51] are able to persist within macrophages and transverse epithelial cells [52]. *B bacilliformis* and *B. australis* are facultative intracellular pathogens found only in a specific region of South America [53] and Australia respectively. The PF07598 family shared between pathogenic *Leptospira* might be shared by other *Bartonella* species that have yet to be sequenced or even identified, such as those recently found in Thailand [54], [55]. The maintenance of multiple members of this paralogous gene family clearly must confer a selective advantage to these pathogens. We performed a meta-analysis of 6 previous studies [12], [56]–[60] that explored transcriptional responses of *L. interrogans* during exposure to host-like physiological conditions (Table S2 in Text S1), and discovered that the expression levels of several of these genes occurs in response to multiple stimuli. This implies that *L interrogans* responds to signals from the host milieu that lead to the alteration of expression of these genes in a differential manner during its infection cycle.

The identification of leptospiral AGC and PF07598 gene family orthologs in specific species of evolutionary distant alpha and delta-proteobacteria was an unexpected and exciting discovery. Given the broad host range of *Leptospira* as well as their environmental persistence, the horizontal gene transfers our findings imply emphasize how the soil context within the unique transmission cycle of *Leptospira* has likely shaped the evolution of pathogenic mechanisms for these bacteria.

Our investigation was not without limitations. The attenuation experiment was done only once. While genes of pathogenetic interest were identified here, whether these mutations occurred stochastically or not remains to be determined. Accumulation of mutations during the attenuation process was not assessed so that step-wise accumulation of mutations could not be attributed to a level of virulence. Proteomic comparisons between *ex-vivo*-isolated and EMJH cultured leptospires were not performed, as our study only focused on gene transcriptional levels, which do not necessarily correlate with protein expression levels. It would be interesting to undertake such *ex-vivo* proteomic investigations in *Leptospira*; especially given the vast transcriptional up-regulation of identified genes upon entry into host tissues. Further investigation remains to define the precise mechanisms of how the identified genes in our study relate to the virulence and pathogenesis of leptospirosis, as a majority of these genes have undiscovered functions.

We show here that a systems biology-pathogenomic approach to infer virulence-related genes in *Leptospira interrogans* identified a notable set of hitherto unstudied genes with both pathogenetic and evolutionary significance, including a putative soluble adenylate/guanylate cyclase (AGC), and a paralogous gene family shared by pathogenic *Leptospira* and the distantly related pathogens *B. bacilliformis*, a human-specific pathogen geographically restricted to the Andes mountains of South America, and *B. australis*, a species currently known to only infect kangaroos. This pathogenomic approach is generalizable beyond prokaryotes and particularly relevant to novel virulence gene identification in any pathogen capable of *in vitro* attenuation. Given the recalcitrant nature of pathogenic leptospires to genetic manipulation, this approach represents an improved method to identify important virulence genes in pathogens whose pathogenesis remains poorly defined by current research strategies, and highlights the extraordinary insights into bacterial pathogenesis and evolutionary biology that large scale genomic sequencing can produce in the context of simple experimentation. These genes will hopefully spur much needed research into the pathogenesis of this neglected disease, but many may also represent rational choices for new vaccine studies.

## Supporting Information

Text S1 **Supporting information.** Includes: **Figure S1.** Validation of 16S rDNA Gene to Normalize Leptospira *In Vivo* Gene Expression. **Figure S2.** Alignment of *Bartonella bacilliformis* and *Leptospira interrogans* serovar Lai anonymous paralog families. **Table S1.** *Leptospira* Species Distribution of Pathogenomically-Discovered Genes. **Table S2.** Differential Expression of Gene Family Members During Exposure of *L. interrogans* to Host-like Conditions. **Table S3.** Primers used for In-vivo RT-qPCR Analysis. **Table S4.** Genome locus tags and GenBank protein sequence accession numbers for *Bartonella bacilliformis* and *Helicobacter* spp. PF07598 family homologs used to construct Figure 5A.
(DOCX)
