# Supplementary material for: Pathogenomic Inference of Virulence-Associated Genes in Leptospira interrogans
Source: PLoS Negl Trop Dis. 2013 Oct 3;7(10):e2468. doi: 10.1371/journal.pntd.0002468 (PMC3789758; doi:10.1371/journal.pntd.0002468)
Supplement: Text S1 — Supporting information. Includes: Figure S1. Validation of 16S rDNA Gene to Normalize Leptospira In Vivo Gene Expression. Figure S2. Alignment of Bartonella bacilliformis and Leptospira interrogans serovar Lai anonymous paralog families. Table S1. Leptospira Species Distribution of Pathogenomically-Discovered Genes. Table S2. Differential Expression of Gene Family Members During Exposure of L. interrogans to Host-like Conditions. Table S3. Primers used for In-vivo RT-qPCR Analysis. Table S4. Genome locus tags and GenBank protein sequence accession numbers for Bartonella bacilliformis and Helicobacter spp. PF07598 family homologs used to construct Figure 5A. (DOCX) [file pntd.0002468.s001.docx]

**Figure S1**

**
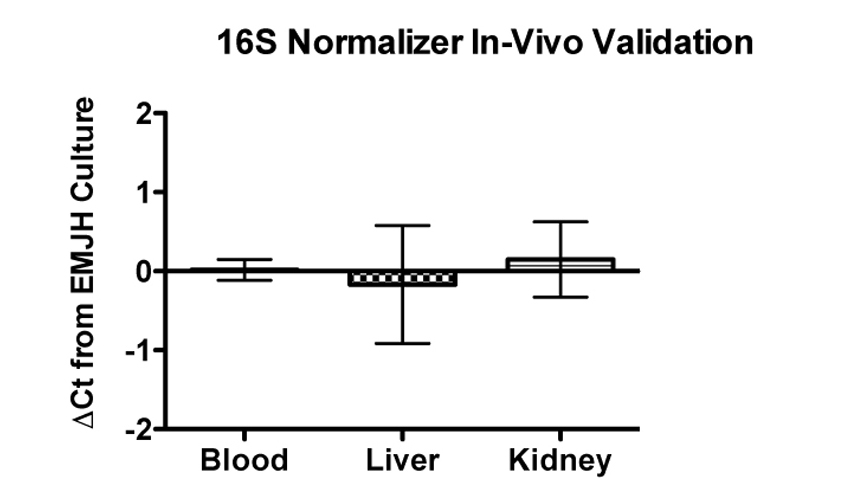
**

**
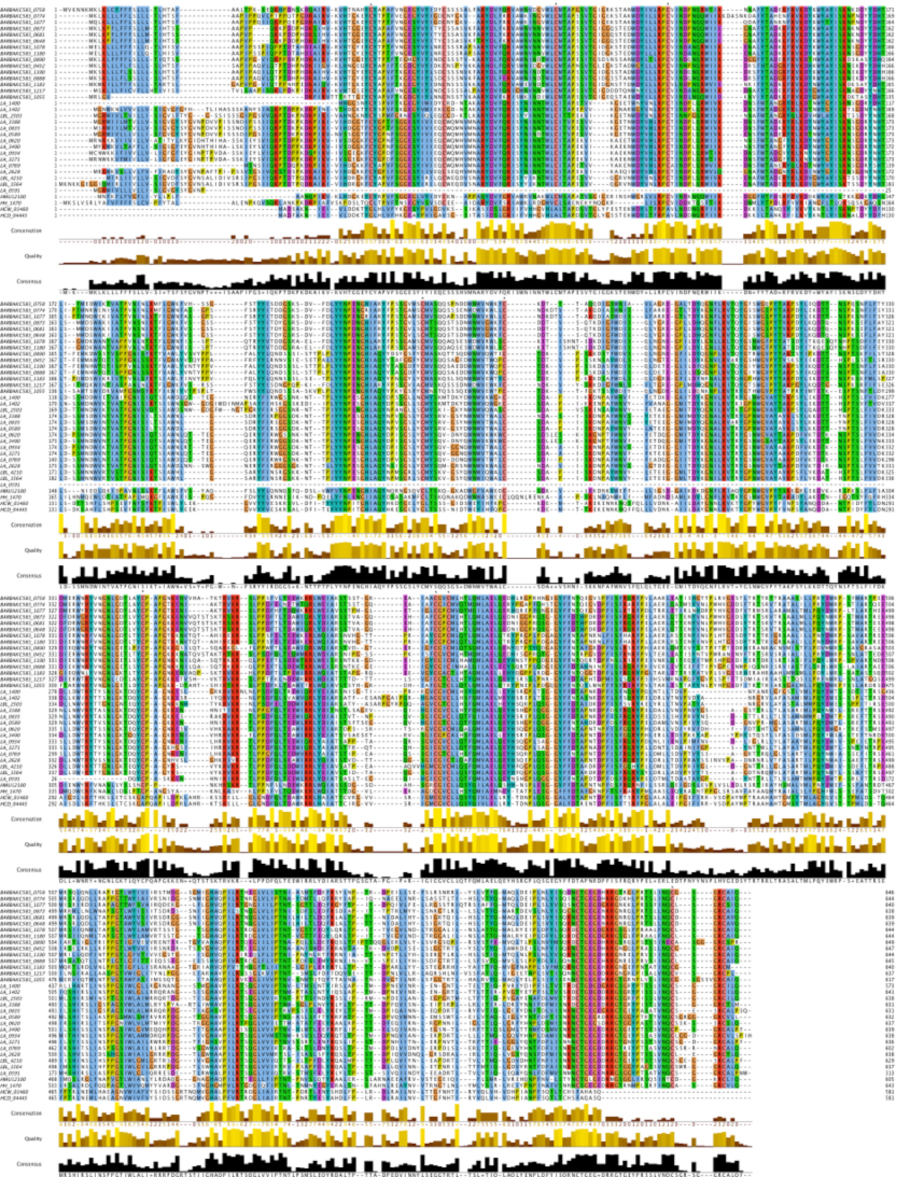
Figure S2**

**Table S1. *Leptospira* Species Distribution of Pathogenomically-Discovered Genes**

**
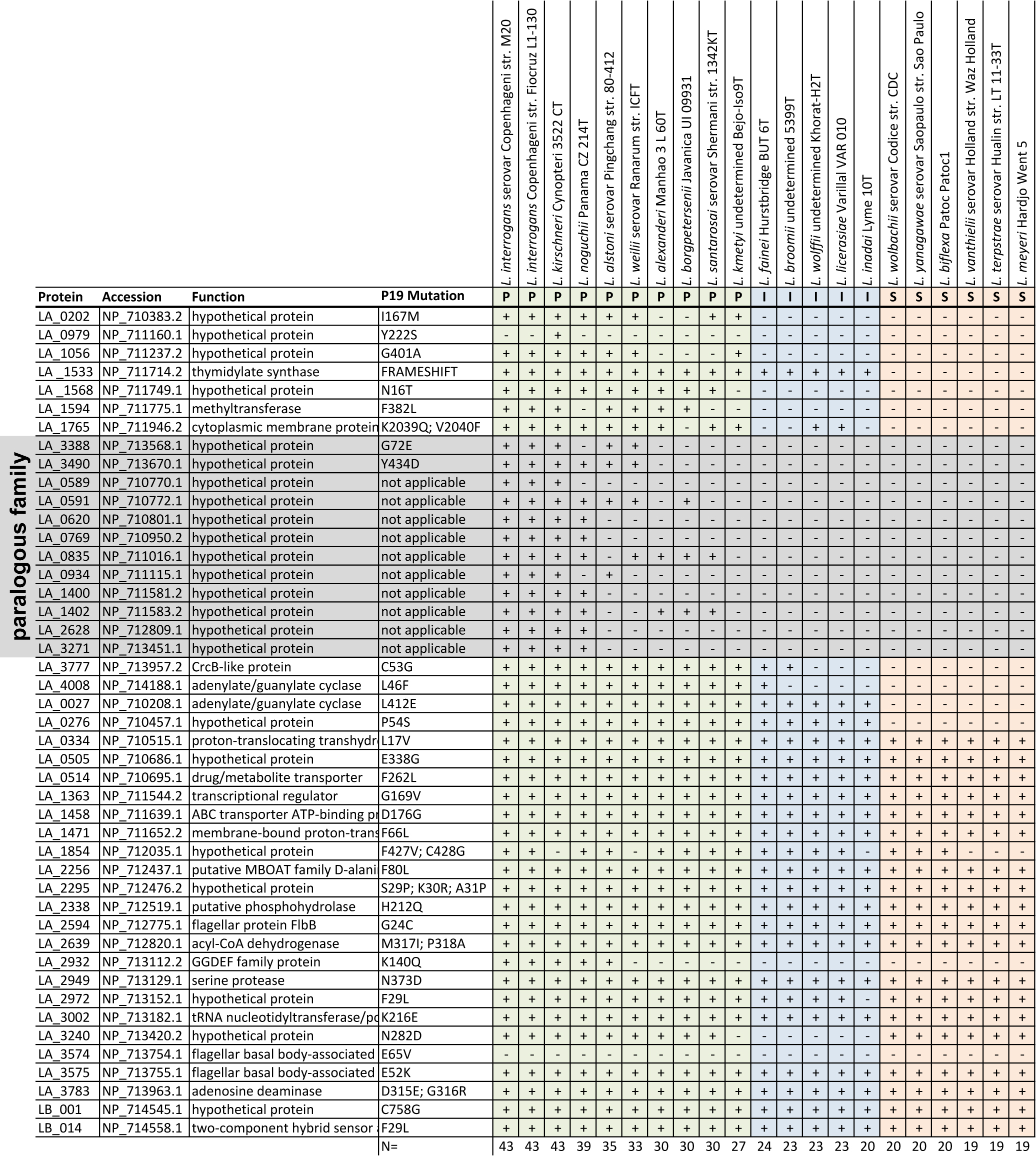
**

**Table S2. Differential Expression of Gene Family Members During Exposure of *L. interrogans* to Host-like Conditions**

|  | **37°C** | **Innate Immune System** | | | **Outer Membrane Proteins**  **37°C** | **Serum** | **Low Iron** | **Osmolarity** |
| --- | --- | --- | --- | --- | --- | --- | --- | --- |
|  |  | **THP-1** | **J774** | **37°C** |  |  |  |  |
| **LA_0835** |  | **↓** |  | **↓** |  |  |  |  |
| **LA_3388** |  |  |  | **↑** |  |  |  |  |
| **LA_0589** |  |  |  |  |  |  | **↑** |  |
| **LA_0591** | **↑** |  |  | **↑** |  |  |  |  |
| **LA_3490** |  |  |  | **↑** |  |  |  |  |
| **LA_0620** |  |  | **↑** | **↑** |  |  |  |  |
| **LA_3271** |  | **↓** |  |  |  |  |  |  |
| **LA_0934** |  |  |  |  |  |  |  | **↑** |
| **LA_1402*** | **↓** | **↑** | **↓** | **↓** | **↓** | **↑** |  | **↑** |
| **LA_1400** |  |  |  |  |  |  |  | **↑** |
| **LA_2628** |  |  |  |  |  |  | **↓** |  |
| **LA_0769** | **↓** | **↓** | **↑** |  |  |  |  |  |

Meta-analysis of 6 studies (refs. ([*12*](#_ENREF_12)*,* [*50-54*](#_ENREF_50))) investigating gene expression by *L. interrogans* during exposure to host-like physiological conditions. Arrows indicate up or down regulation of at least 2-fold as compared to EMJH medium controls. * = protein detected in the outer membrane fraction (59) indicating that it may be surface-expressed.

**Table S3.** Primers used for In-vivo RT-qPCR Analysis

| Gene | Forward Primer (5’-3’) | Reverse Primer (5’-3’) |
| --- | --- | --- |
| LA_0202 | CTTTCCTTGCGGCTTAGGAACGTA | GGCCCAACTCCATAAGGGTCCGA |
| LA_3490 | TCCCCTGCTGGAAGCATTTGGT | TGAGACAGCTCTGTGGTGGGT |
| LA_3388 | AGGTTCTCCCGCCGGAAGTGT | ACATTCAATGGCAGCCCAGACCA |
| LA_1056 | CGATCGGCGGGAAAAGTTCGGA | TAAAACCGGCCGCCGCCATAC |
| LA_1568 | GCCCGTGAGTGCTCTCGGATA | GGCGGCCTCTGGAAATCGGG |
| LA_1533 | TGGATAGTTGGCACGGGCCTT | TCCCACCCGAACTCGACGGA |
| LA_4008 | GTGCTCCCGCAATCACGGCT | GAGACCGCCGAAGAGGCTGC |
| LA_1594 | GGTGCGGGCAGTTTTGTTGTGG | TGTCGCGTCTTCCGCGGTTT |
| LA_3777 | TGGCTTCTGGTTTTTGCGGAGGA | GCGAAAGCAAAACCAATTCCACCA |
| LA_1765 | CGCCGCAAAAGAAAGGAGGAAGT | TCCGAGTAAAGCCCACCACCAGT |
| LA_0979 | GGCGCATGGGGCGAAAAGGA | AGGCAATTGCGTTCCTTGGGCT |
| 16S | GAGTTTGGGAGAGGCAAGTGGAATCCA | CGCTTTCGTGCCTCAGCGTCAGTTTTA |

| Gene | Forward Primer (5’-3’) | Reverse Primer (5’-3’) |
| --- | --- | --- |
| LA_0835 | ACAGGAATTGTACCGGGGAAGGGGA | GGTAGTGCGCACCTGCTTTCCGAGC |
| LA_0589 | ACGGGAGGTCATGCCGTTCCAA | ACCGGGTCCGTAGTAGGTGTTGC |
| LA_0591 | TGCGGAGTTTGTCTGCTTCAGACCT | AGCCGCATCCGTCAGCATCGTGT |
| LA_0620 | ACGAATGGGGGCGCACTCGTGA | CCGCATGCCCTCCTCTCGTTCCA |
| LA_3271 | GGCAATACTGCGATGGCGGCG | GTCTGAAGTTTTCAAGTGTGCGCGA |
| LA_0934 | CCCCCGGAAGTTTTTGGTTGGCT | TCCAGTGACACCGAACGCCAGT |
| LA_1402 | TGAATGGGAGTCCTCTTCTCCGCT | AGGAACGGCATGCCCCGAAAT |
| LA_1400 | CTTGGGGGCATGCCGTTCCCA | CCGGCTGTATGGTTGTAAGTTCCGT |
| LA_2628 | GGGTGAAGACCGTGGCTACTCCCG | ACCGGAACCTCCCCCACGAAT |
| LA_0769 | CGTACGGATGGAGTTAGTGGTTGGC | TGTCAAGCGACGTCAAAGAGGCCC |

**Table S4. Genome locus tags and GenBank protein sequence accession numbers for *Bartonella bacilliformis* and *Helicobacter* spp. PF07598 family homologs used to construct Figure 4B.**

| Locus Tag ID | GenBank Protein Accession Number |
| --- | --- |
| ***Bartonella bacilliformis*** |  |
| BARBAKC583_0452 | YP_988769.1 |
| BARBAKC583_0648 | YP_988951.1 |
| BARBAKC583_0681 | YP_988982.1 |
| BARBAKC583_0758 | YP_989055.1 |
| BARBAKC583_0774 | YP_989068.1 |
| BARBAKC583_0873 | YP_989160.1 |
| BARBAKC583_0888 | YP_989172.1 |
| BARBAKC583_0890 | YP_989175.1 |
| BARBAKC583_1055 | YP_989329.1 |
| BARBAKC583_1077 | YP_989345.1 |
| BARBAKC583_1078 | YP_989346.1 |
| BARBAKC583_1180 | YP_989438.1 |
| BARBAKC583_1183 | YP_989441.1 |
| BARBAKC583_1190 | YP_989445.1 |
| BARBAKC583_1217 | YP_989469.1 |
|  |  |
| ***Helicobacter* spp.** |  |
| *H. cetorum* HCW_01460 | YP_006222207.1 |
| *H. hepaticus* HH1470 | NP_861001.1 |
| *H. mustelae* HMU12100 | YP_003517189.1 |
